# Genomic Islands Confer Heavy Metal Resistance in *Mucilaginibacter kameinonensis* and *Mucilaginibacter rubeus* Isolated from a Gold/Copper Mine

**DOI:** 10.3390/genes9120573

**Published:** 2018-11-23

**Authors:** Yuan Ping Li, Nicolas Carraro, Nan Yang, Bixiu Liu, Xian Xia, Renwei Feng, Quaiser Saquib, Hend A Al-Wathnani, Jan Roelof van der Meer, Christopher Rensing

**Affiliations:** 1 Institute of Environmental Microbiology, Fujian Agriculture and Forestry University, Fuzhou 350002, China; li343000@126.com (Y.P.L.); cindyyn1118@163.com (N.Y.); liu_bixiu@163.com (B.L.); 2 Department of Fundamental Microbiology, University of Lausanne, Lausanne 1015, Switzerland; nicolas.carraro@unil.ch (N.C.); janroelof.vandermeer@unil.ch (J.R.v.d.M.); 3 State Key Laboratory of Agricultural Microbiology, College of Life Science and Technology, Huazhong Agricultural University, Wuhan 430070, China; xiaxian@webmail.hzau.edu.cn; 4 Zoology Department, College of Sciences, King Saud University, P.O. Box 2455, Riyadh 11451, Saudi Arabia; qsaquib@ksu.edu.sa; 5 Department of Botany & Microbiology, College of Sciences, P.O. Box 2455, Riyadh 11451, Saudi Arabia; wathnani@ksu.edu.sa; 6 Key Laboratory of Urban Environment and Health, Institute of Urban Environment, Chinese Academic of Sciences, 361021 Xiamen, China

**Keywords:** *Mucilaginibacer rubeus*, *Mucilaginibacter kameinonensis*, genomic island, evolution, heavy metal resistance, draft genome sequence, CTnDOT

## Abstract

Heavy metals (HMs) are compounds that can be hazardous and impair growth of living organisms. Bacteria have evolved the capability not only to cope with heavy metals but also to detoxify polluted environments. Three heavy metal-resistant strains of *Mucilaginibacer rubeus* and one of *Mucilaginibacter kameinonensis* were isolated from the gold/copper Zijin mining site, Longyan, Fujian, China. These strains were shown to exhibit high resistance to heavy metals with minimal inhibitory concentration reaching up to 3.5 mM Cu^(II)^, 21 mM Zn^(II)^, 1.2 mM Cd^(II)^, and 10.0 mM As^(III)^. Genomes of the four strains were sequenced by Illumina. Sequence analyses revealed the presence of a high abundance of heavy metal resistance (HMR) determinants. One of the strain, *M. rubeus* P2, carried genes encoding 6 putative P_IB-1_-ATPase, 5 putative P_IB-3_-ATPase, 4 putative Zn^(II)^/Cd^(II)^ P_IB-4_ type ATPase, and 16 putative resistance-nodulation-division (RND)-type metal transporter systems. Moreover, the four genomes contained a high abundance of genes coding for putative metal binding chaperones. Analysis of the close vicinity of these HMR determinants uncovered the presence of clusters of genes potentially associated with mobile genetic elements. These loci included genes coding for tyrosine recombinases (integrases) and subunits of mating pore (type 4 secretion system), respectively allowing integration/excision and conjugative transfer of numerous genomic islands. Further in silico analyses revealed that their genetic organization and gene products resemble the *Bacteroides* integrative and conjugative element CTnDOT. These results highlight the pivotal role of genomic islands in the acquisition and dissemination of adaptive traits, allowing for rapid adaption of bacteria and colonization of hostile environments.

## 1. Introduction

Heavy metals (HMs) have a dualistic impact on living organisms. On the one hand, metal ions are essential for numerous biological processes mandatory for cellular activity, including homeostasis, enzyme activity, and protein functionality [1]. On the other hand, when present in excess in the environment, HM can have toxic effect hindering diverse cellular processes and thus cellular life.

Heavy metal pollution has been part of Earth’s history as it can originate from natural processes such as volcanic eruption. Recent (over)industrialization and exploitation of Earth resources worldwide has accelerated HM release into the environment and led to high levels of water, air, and soil pollution. Especially, mine exploitation for metal extraction is one of the most important sources of heavy metal pollution [2]. This comes not only from excavating deep-buried HMs to be exposed to the surface, but also from extraction protocols that often rely on the use of other contaminants, including HMs [2].

Beyond its effects on people, HM toxicity was shown to have profound impacts on microbial communities, including fungi and bacteria [2]. Heavy metals were shown to have critical consequences on bacterial viability due to their pleiotropic effect on cellular processes. Excess of HM can disrupt the cell membrane, damage nucleic acids and proteins, impair enzymatic activities, and inhibit key processes such as transcription [1]. The presence of HM pollution exerts a high selective pressure on microbial communities, reducing their diversity, biomass, and activity, thus strongly impacting the biological activity of polluted environments [3].

In order to cope with the presence of elevated concentration of HMs, a myriad of bacterial genetic programs has been selected encoding functions that allow efflux and/or sequestration of HMs, and modification to inactivate or reduce reactivity of certain metal ions. The main mechanism to resist toxicity of HMs is efflux [1]. Important classes of HM transporters include P_IB_-type ATPases and cation diffusion facilitators (CDF). Both types of transporters translocate HM ions from the cytoplasm across the cytoplasmic membrane into the periplasm [4]. In the context described here with microbes having to handle very high external concentrations of HMs, P-type ATPases are much more relevant since they are much more powerful using ATP to pump HMs against their concentration gradient out of the cytoplasm [5]. In addition, HMs are translocated from the periplasm across the outer membrane into the extracellular space by resistance-nodulation-division (RND)-type transport systems. These multicomponent transporters of the RND type contain 3 RND transport proteins, 6 membrane fusion proteins (MFPs), and 3 outer membrane factor (OMF) proteins. The fascinating transport mechanism of the RND-type transport complex has been described in detail [4]. P_IB_-type ATPases and RND-type transport systems were described as being the most important systems to confer a high HM resistance (HMR).

Bacteria also show an astonishing capability to spread HMR genes within bacterial communities via horizontal gene transfer. Dissemination of genetic material conferring HMR is frequently associated with conjugative plasmids, genomic islands, and transposons [6]. Conjugative plasmids are extrachromosomal replicative entities able to transfer from a donor cell toward a recipient cell by conjugation [7]. Conjugative plasmids have been recognized as major contributors for the spread of adaptive traits such as antibiotic resistance, new metabolic capacities, and HMR [8]. Conjugative plasmid-borne HMR is associated with occurrence of large clusters of HMR genes that can span over several kb [9,10,11,12,13,14,15]. Portions of genomic DNA called genomic islands (GIs) were also shown to play a pivotal role into the horizontal dissemination of genetic material [16]. Although the mechanisms underlying the mobility of some GIs remain obscure, current knowledge describes different strategies that ultimately rely on conjugative transfer [17,18]. GI-associated HMR was described in *Enterobacteriaceae* and *Shewanellaceae* [19], *Listeria monocytogenes* [20], and *Acinetobacter baumannii* [21]. Also, HMR was shown to be conferred by an IncC-dependent mobilizable genomic island SGI1 variant called SGI1-K in *Salmonella enterica* [22,23,24]. Transposons are genetic entities able to move intra-molecularly (on the same replicon) or inter-molecularly (between different replicons) [25]. Most transposons can hitchhike by integrating into a conjugative plasmid or a GI for intercellular mobility. Transposons conferring HMR were described to be in association with other mobile genetic elements [13,22].

In this study, we describe the isolation and characterization of four heavy metal-resistant *Mucilaginibacter* strains isolated from a gold/copper mine in China. Genomes of these strains were sequenced and further in silico analysis revealed a high number of heavy metal resistance determinants. Moreover, at least part of these HMR gene clusters were shown to be potentially mobile as they are in the close vicinity of the core region of putative integrative and conjugative elements (ICEs).

## 2. Materials and Methods

### 2.1. Bacterial Isolation

Strains *Mucilaginibacter rubeus* P1, P2, and P3 were isolated from samples collected at 5–10 cm below the surface of a soil located near a waste water treatment dam of a copper-gold mine, and *Mucilaginibacter kameinonensis* P4, was isolated from a hillside with little human activity within the gold and copper mine (Zijin mining) in Longyan city of Fujian province, China (Table 1). After serial dilutions with 0.85% NaCl, the soil sample was spread on R2A (DSM medium 830) agar plates containing 2 mM CuSO_4_·5H_2_O. After incubation at 28 °C for 1 week, the strains were isolated and later stored at −80 °C in 20% glycerol (*w*/*v*).

### 2.2. Taxonomic Analysis

Strains were incubated at 28 °C for 24 h on R2A agar plates. As described in Brosius et al. [26], the universal primer pair 27F/1492R was used to amplify 16S sequences and the amplified PCR product was subsequently sequenced [26]. PCR products were sequenced by Biosune Company (Fuzhou, China) using the Sanger method. Based on the EzTaxon database (http://eztaxon-e.ezbiocloud.net) [27], pairwise sequence similarity and phylogenetic neighbors of the sequences of each individual strain (1382–1432 bp) were obtained through BLAST searches. In total, 19 *Mucilaginibacter* strains with publicly available 16S ribosomal RNA (rRNA) gene sequences were selected, with *Pedobacter africanus* DSM 12126T (AJ438171) as an out-group, to do the alignment via Mega 7.0 software [28]. A Neighbor-joining (NJ) tree was generated and the Kimura’s two-parameter model was used to calculate evolutionary distances [29], and bootstrap analysis with 1000 replications was conducted to obtain confidence levels of the branches [30].

### 2.3. Determination of the Minimal Inhibitory Concentration

To determine the level of resistance to various metals of all strains, *M. rubeus* P1, P2, and P3 and *M. kameinonensis* P4 were grown on Cu, As, Cd, and Zn agar plates containing different Cu^(II)^, Zn^(II)^, As^(III)^, and Cd^(II)^ concentrations to determine the minimal inhibitory concentration (MIC). The different R2A plates contained 0–10.0 mM of copper or arsenic, with 0.5 mM increments, 0–30.0 mM with 1.5 mM increments in case of zinc, and 0–2.0 mM cadmium with the increments being 0.2 mM. 1M CuSO_4_·5H_2_O, ZnCl_2_, NaAsO_2_, and CdCl_2_·5H_2_O stock solutions were prepared and stored after filtration through a 0.22 µm filter.

### 2.4. Cell Morphology and Flagella Observation

Overnight cultures of strains *M. rubeus* P1, P2, and P3 and *M. kameinonensis* P4 were inoculated into 50 mL of R2A medium at 28 °C with 180 rpm shaking. After 24 h of growth with shaking, cells were centrifuged (1000× *g*, 10 min, 4 °C) and observed under scanning electron microscopy (SEM). Cells were harvested and washed three times with cold (4 °C) phosphate buffered saline (0.2 M PBS, pH 7.2). Fixation was performed with 2.5% glutaraldehyde (24 h, 4 °C). Fixed cells were dehydrated through a series of alcohol dehydration steps (30%, 50%, 70%, 85%, 95%, and 100%) and finally freeze dried and sputter coated. The samples were then viewed using a scanning electron microscope JSM-6390 SEM (JEOL, Tokyo, Japan).

### 2.5. Growth Conditions Optimization

To optimize NaCl concentration and pH of the medium for growth of the *Mucilaginibacter* strains, 50 μL precultures were added to 5 mL R2A liquid medium supplemented with 0–3% NaCl at pH 7, or to R2A without any NaCl and with pH set to the range between pH 2–11. Cultures were incubated at 28 °C for 7 days, after which culture turbidities optical density (OD) at 600nm were evaluated. Anaerobic growth was tested by incubating R2A plates in an anaerobic chamber at 28 °C for 1 week. Optimal growth temperature was tested in the incubator on R2A agar plates at temperatures between 4 to 40 °C for 1 week.

### 2.6. Genomic DNA Extraction

Genomic DNA (gDNA) was extracted by using a TIANamp Bacteria DNA Kit (Tiangen Biotech, Beijing, China) from cultures grown on R2A. The quantity and purity of gDNA were assessed using an UV spectrophotometry (Nanodrop ND-1000, J & H Technology Co., Ltd. Wilmington, USA). Genomic DNA with OD260/280 value higher than 1.80 was selected and examined on agarose gel electrophoresis (0.8%). Samples containing more than 25 μg of intact gDNA (fragment size > 20 kb) were sent out for whole-genome sequencing.

### 2.7. Whole-Genome Sequencing

Whole-genome shotgun sequencing was preformed using an Illumina HiSeq X Ten System provided by Vazyme Biotech Co., Ltd. (Nanjing, China). The DNA library was constructed using the Illumina V3 VAHTS Universal DNA Library Prep Kit according to the VAHTS Universal DNA sample preparation protocol (Illumina, Santiago, USA). The insert size was 300 bp for all strains, and 16,980,768, 18,531,104, 18,306,636, and 20,005,292 read-pairs and 2.86, 3.12, 3.09, and 3.37 Gb of raw data were obtained for strains *M. kameinonensis* P4 and *M. rubeus* P1, P2, and P3, respectively.

### 2.8. De novo Genome Assembly and Annotation

Illumina reads were quality-filtered, trimmed, and de novo assembled with default settings using CLC Genomic Workbench 11.0 (QIAGEN, Hilden, Germany). The draft genome sequences were annotated by NCBI PGAP, and are accessible under GenBank numbers QEYR0000000, QFKW0000000, QFKV0000000, and QFKU0000000 for *M. kameinonensis* P4 and *M. rubeus* P1, P2, and P3, respectively. *M. kameinonensis* P4 generated 78 contigs with an n50 value of 350.607 bp. *M rubeus* P1 generated 158 contigs with an n50 value of 139.339 bp. *M rubeus* P2 generated 118 contigs with an n50 value of 132.524 bp. *M rubeus* P3 generated 107 contigs with an n50 value of 148.541 bp.

### 2.9. TraG Proteins Phylogenetic Analyses

Molecular phylogenetic analysis of TraG proteins was performed using MEGA6 [28]. The 807- to 850-amino acid sequences of TraG proteins were recovered from genome sequences of *Mucilaginibacter* isolated in this study. The corresponding sequence in CTnDOT (TraG_DOT_ accession number: AAG17832.1) was added to the dataset as an outgroup. Analyses were computed using an amino acid alignment generated by MUSCLE [31]. The evolutionary history was inferred by using the Maximum Likelihood method based on the Jone, Taylor and Thornton (JTT) matrix-based model [32]. Initial tree(s) for the heuristic search were obtained by applying the NJ method to a matrix of pairwise distances estimated using a JTT model. A discrete Gamma distribution was used to model evolutionary rate differences among sites (five categories (+G, parameter = 2.9848)). The analysis involved 18 amino acid sequences. All positions with less than 95% site coverage were eliminated, providing a total of 716 positions in the final dataset.

## 3. Results and Discussion

### 3.1. Isolation of Four Heavy Metal-Resistant Mucilaginibacter

We intended to isolate heavy metal resistant strains from the ZiJin copper-gold mine to gain insights into how bacterial strains adapt to high concentrations of HMs. We recovered four strains that were morphologically similar with a high tolerance to a number of HMs.

**Figure 1:**
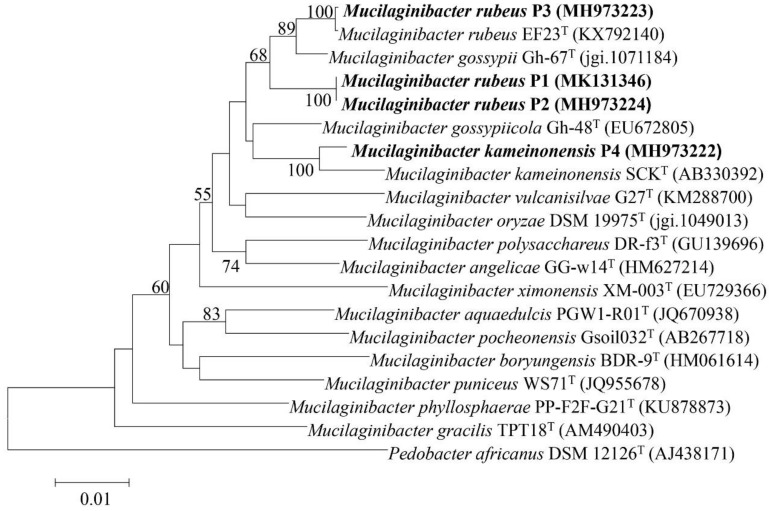
Neighbour-joining phylogenetic tree constructed based on the 16S ribosomal RNA (rRNA) gene sequences from the draft genome sequence showing the phylogenetic relationships between strains *Mucilaginibacter rubeus* P1, P2 and P3 and *Mucilaginibacter kameinonensis* P4 and other species in the genus *Mucilaginibacter*. Values indicate percentages of identical branching in 1000 bootstrappings. The sequence of *Pedobacter koreensis* WPCB189T was used as an out-group. Bar, 0.01 substitutions per nucleotide position.

Based on phylogenetic analysis (NJ) of the 16S rRNA gene three strains (P1, P2, and P3) were closely related to *M. rubeus* EF23^T^ (98.34–99.93%) and *M. gossypiicola* Gh-67^T^ (98.12–99.01 %). The fourth strain (P4) grouped closely with *M. kameinonensis* SCK^T^ (98.8 %) (Figure 1). All strains belonged to the *Sphingobacteriaceae* family in the class *Sphingobacteriia*.

### 3.2. Phenotypic Characterization of Mucilaginibacter Strains Uncovered Multiple Heavy Metal Resistances

The HM concentration of the soil is extremely high, even the lowest concentration of total Zn, As, Cd, and Cu was found to be 49.27, 1.43, 1.19, and 18.37 mg·kg^−1^, respectively. The MICs of the four strains reached up to 3.5 mM Cu^(II)^, 21 mM Zn^(II)^, 1.2 mM Cd^(II)^, and 10.0 mM As^(III)^. Strain *M. kameinonensis* P4 displayed higher Cd resistance compared to strains *M. rubeus* P1, P2, and P3 (Table 2). Related, not heavy metal resistant *Mucilaginibacter pedocola* sp. TBZ30^T^, cultured under similar conditions displayed MICs of 0.4 mM Cu^(II)^, 3 mM Zn^(II)^, 0.2 mM Cd^(II)^, and 0.2 mM As^(III)^ [33]. Such high resistance to multiple HMs as reported here has therefore not been observed before in the genus *Mucilaginibacter* [33].

Strains *M. rubeus* P1, P2, and P3 and *M. kameinonensis* P4 formed a light orange or pink, moist, circular, and convex colony with smooth margins on R2A agar plates. All strains were Gram-negative and aerobic. Growth of strains was observed at 4–30 °C (optimum, 28 °C). Optimal growth occurred in absence of further NaCl, but the strains could still grow in R2A with up to 1.5% NaCl added. These characteristics are consistent with description of the genus *Mucilaginibacter* [34,35,36]. Medium pH for optimal growth (~pH 5.0) and pH tolerance (pH 5.0–9.0) varied slightly between the four strains (Table 3).

### 3.3. Mucilaginobacter Strains Exhibit an Arsenal of Genetic Determinants to Deal with High Concentrations of Heavy Metals

To gain insight in the genetic basis of how the four strains were able to deal with these high HM concentrations, we determined draft genome sequences. Draft genomes were automatically annotated through RAST (Rapid Annotation using Subsystem Technology) database (http://rast.nmpdr.org/). Based on inferred protein homologies, between 6 and 16 putative P_1B_ type-ATPase [37] were encoded in the four genomes (Table 4). All strains further encoded a variety of putative RND type metal transporter systems of the CzcCBAD type. Three strains further encoded putative CusCBA Cu^(I)^ translocating RND-type transport systems, except *M. kameinonensis* P4 (Table 3). Multiple genes for putative multicopper oxidases were found on the different genomes, which may constitute the basis for the observed copper resistance (Table 3). Genes for putative multicopper oxidases were only taken into account if they were located adjacent to genes encoding P_1B_ type Cu^(I)^ translocating P-type ATPase. Finally, between 2 and 4 putative *ars* operons (*arsNCR, acr3, arsMCR*) were among the *Mucilaginibacter* genomes (Table 3). The higher number of *ars* operons in strain P1 and P2 genomes correlated to their high MICs on As^(III)^ (10.0 and 9.0 mM, respectively).

The number of HMR determinants in *Mucilaginibacter* genomes was unusually high, even in comparison to the well-known HM-resistant strain *Cupriavidus metallidurans* CH34 [5,38,39] (Table 4), suggesting a strong selection for HM resistance in their natural living environment. The HM resistance determinants are often clustered together, and often located adjacent to *tra* genes. They could be identified on many different contigs.

### 3.4. Heavy Metal Resistance Is Associated with CTnDOT-Related Genomics Islands

**Figure 2:**
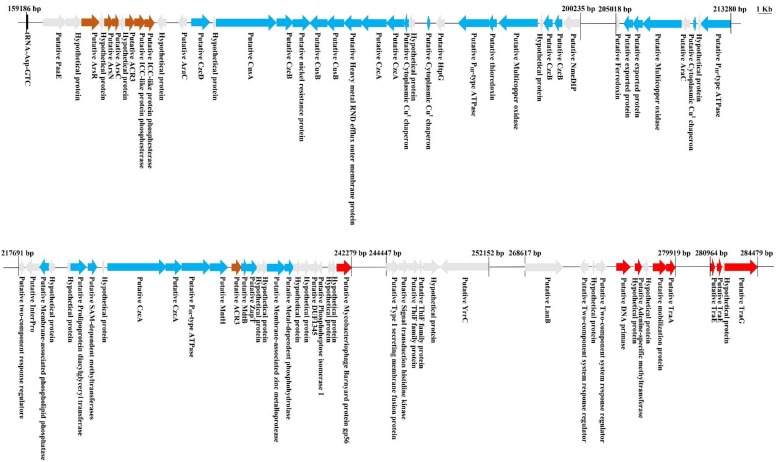
Representative putative genomic island carrying genes encoding HM determinants in contig 26 of *M. Rubeus* P2. Genomic analysis was performed via RAST (http://rast.nmpdr.org/). Genes encoding determinants related to metalloid (Arsenical and Antimony) resistance are highlighted in maroon, metal (Copper/Cobalt/Cadmium/Zinc/Lead/Mercury) resistance in blue, and the genes encoding putative transfer functions in red. Genes encoding hypothetical proteins and unknown functions are highlighted in gray.

Tolerance to HMs is frequently acquired by horizontal transmission among and between bacterial populations. Given the important size of HMR clusters identified in *Mucilaginibacter* genomes (up to 150 kb), we wondered whether some might be encompassed by GIs (Figure 2). We examined the close vicinity of the HMR clusters for the hallmark of conjugative systems, also known as Type 4 secretion systems (T4SSs) [40]. T4SSs have been classified based on their VirB4 protein, a ubiquitous constituent of conjugative systems [41]. A total of 17 genes encoding VirB4 proteins (*traG*) exhibiting sizes between 807 to 850 amino acids were identified on the 4 genomes, most of them in the close proximity of HMR clusters: 6 in *M. rubeus* P1 (contigs 1, 4, 24, 29, 42, 55), 6 in *M. rubeus* P2 (contigs 24, 26, 29, 32, 34, 42), 2 in *M. rubeus* P3 (contigs 5 and 11), and 3 in *M. kameinonensis* P4 (contigs 6, 9, and 35). 

**Figure 3:**
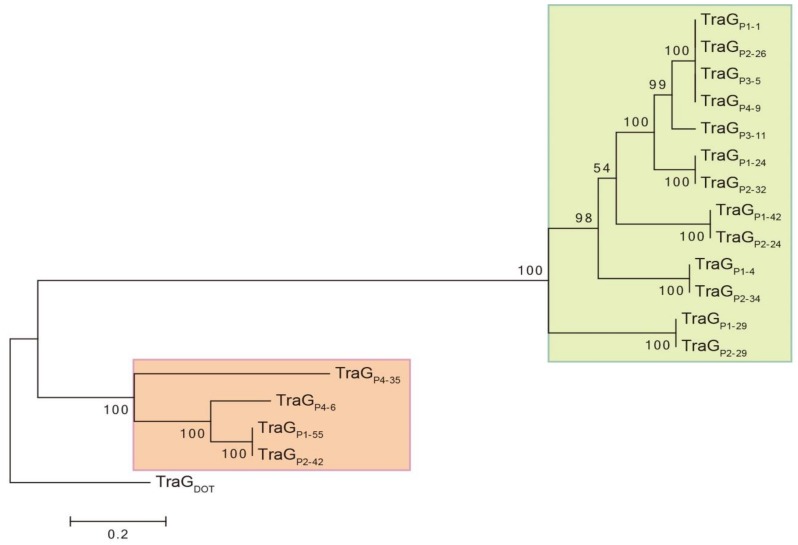
Molecular phylogenetic analysis of TraG proteins of putative conjugative genomic islands (GIs) of *Mucilaginibacter*. The evolutionary history was inferred by using the Maximum Likelihood method based on the JTT matrix-based model [32]. The percentage of trees in which the associated taxa clustered together is shown next to the branches. The tree is drawn to scale, with branch lengths measured in the number of substitutions per site. Evolutionary analyses were conducted in MEGA6 [28]. Initial alignment of sequences was performed using Muscle for the presented tree. An identical tree with minor changes in bootstrap values was obtained using ClustalW for alignment. The VirB4 subunit of MPFB T4SS is named TraG [41]. For convenience and consistence in TraG protein identification, nomenclature is as follows: TraGPX-Y, where X is the strain number and Y the contig carrying the gene coding for TraG. TraG CTnDOT (TraGDOT) accession number: AAG17832.1.

TraG proteins of the putative conjugative GIs identified in *Mucilaginibacter* genomes were compared to the 838-amino acid TraG of CTnDOT (TraG_DOT_) and showed 32 to 53% of identity over 94 to 98% of their amino acid sequence. The evolutionary history of TraG proteins was inferred using TraG_DOT_ as an outgroup (Figure 3). Two strongly supported clades were delineated, suggesting that they belong to two distinct lineages (Figure 3, green and red boxes). As expected, each one of the TraG proteins of strain *M. rubeus* P1 grouped with one TraG protein from strain *M. rubeus* P2, confirming that these identical strains contain the same 6 elements. More interestingly, TraG_P1-1_, TraG_P2-26_, TraG_P3-5_, and TraG_P4-9_ grouped together, and their gene sequences were identical.

Closer analysis revealed the presence of genes coding for other T4SS subunits adjacent to each one of the *traG* genes. This grouping of *tra* genes may be regarded as a conjugation module, i.e., genes and sequences implicated in the same biological process [42]. In particular the regions including *traG_P1-1_, traG_P2-26_, traG_P3-5_*, and *traG_P4-9_* were 100% identical across a circa 150-kb region, including an about 75-kb cluster coding for multiple HMR. The organization of these putative conjugation modules resembles the one encoded in CTnDOT, a protypical ICE of *Bacteroides* [41,43]. As in CTnDOT, putative conjugative modules encoded by GIs of *Mucilaginibacter* thus belong to the mating pair formation (MPF) category B (MPFB) [41]. Also, most of the putative conjugative modules identified in *Mucilaginibacter* strains are in the proximity of genes encoding a putative tyrosine recombinase related to IntDOT, the integrase of CTnDOT [44]. Moreover, the *Mucilaginibacter* GIs carried a gene predicted to encode a RteC-like protein reminiscent of the CTnDOT regulation system [45,46]. *Mucilaginibacter* GIs are thus likely to be ICEs, whose maintenance relies on integration into the chromosome and dissemination depends on its excision from the chromosome as a circular element that would transfer by conjugation [17,47,48]. The presence of at least one identical contiguous region over 150 kb (represented by the *traG_P1-1_* gene) in the four different *Mucilaginibacter* recovered strains suggest active mobility and recent transfer of this GI.

Since the draft genomes were not completely curated to a single contiguous scaffold, we could not confidently delimit the boundaries of the putative conjugative GIs. As a matter of fact, a single GI might be spread over multiple contigs, or could be a defective element lacking flanking or internal parts of the original GI. Also, IntDOT was reported to not require strict homology between the recombining sites in contrast to the majority of tyrosine recombinases [46,49]. The integration/excision is, in that case, site-selective rather than site-specific, strongly impairing the precise identification of the right and left attachment sites (*attR* and *attL*, respectively).

## 4. Concluding Remark

This work allowed the isolation and characterization of four heavy metal-resistant *Mucilaginibacter* strains recovered from polluted soil of gold mines. Sequencing of genomic content allowed inspection of HMR loci into the chromosome of these strains and their close association with loci coding for conjugation of CTnDOT-related GIs. Further genome closure and experimental investigation should allow testing the functionality of such putative ICEs found in the *Mucilaginibacter* strains. Notably, phenotypes such as their ability to excise from the chromosome and their capability to transfer toward a new host by conjugation will be monitored. In particular the 150-kb (at least) conserved putative ICE present in the four strains is an interesting candidate, likely to be functional given its complete identity among all four strains. Protein BLAST using TraG of this conserved element and search *Mucilaginibacter* genomes did not give other perfect hits, suggesting that presence of this GI is restricted to sampling locations of this study (Table 1). Increasing availability of fully sequenced genomes should allow further data meaning in order to evaluate the abundance of specific GIs in *Mucilaginibacter* and predict their functionality.

The presence of putative CTnDOT-related ICEs into genomes of *Mucilaginibacter* strains does not seem uncommon. Protein BLAST analysis using TraG_DOT_ as query and searching *Mucilaginibacter* genomes gave multiple hits with identity ranging from 70% over 99% of aa sequence, down to 30% over 79% of aa sequence (considering 96 hits with more than 75% of coverage). This observation highlights the presence of multiple putative CTnDOT-related GIs populating *Mucilaginibacter* genomes, most likely playing a key role in their genome evolution and adaptation. One can speculate that such GIs may be involved in conferring specific capabilities such as the ability to degrade pectin, xylan, and laminarin of *M. paludis* and *M. gracilis* [50] plant growth promotion capacity conferred by *M. gossypii* and *M. gossypiicola* [51], or yet to be discovered adaptive functions that may be conferred by such GIs. Further *in silico* analyses may reveal interesting features of such putative ICEs considering accessory functions they could confer, or functionality of their recombination, conjugation, or regulatory systems.

This exploratory work on HMR GIs of *Mucilaginibacter* together with other research done on *Mucilaginibacter* species so far constitutes a solid ground for future experimental research aiming at developing molecular tools. Such tools would greatly facilitate further investigation of *M. rubeus* and *M. kameinonensis* biology and could likely be extended to other *Mucilaginibacter* species.

## Figures and Tables

**Table 1:** Characteristics of the heavy metal (HM)-contaminated soil from where the strains were isolated.

	*Mucilaginibacter kameinonensis* P4	*Mucilaginibacter rubeus* P3	*M. rubeus* P2	*M. rubeus* P1
Altitude (m)	216	192	192	192
Longitude	N25°09.719′	N25°09.724′	N25°09.724′	N25°09.724′
Latitude	E116°23.258′	E116°23.258′	E116°23.258′	E116°23.258′
pH	6.64	5.52	6.32	6.32
Water content	9.38%	6.41%	7.05%	7.05%

**Table 2:** Minimal inhibitory concentration (MIC) of strains to Zn^(II)^, As^(III)^, Cd^(II)^, and Cu^(II)^ and respective concentrations of the HM in the soil where the strains were isolated from.

Metals	*M. kameinonensis* P4	*M. rubeus* P3	*M. rubeus* P2	*M. rubeus* P1	*M. pedocola* sp. TBZ30^T^
Zn^(II)^/mM	10.5	21.0	10.5	21.0	3.0
As^(III)^/mM	3.5	4.5	9.0	10.0	0.2
Cd^(II)^/mM	1.2	0.2	0.4	0.4	0.2
Cu^(II)^/mM	3.5	3.5	3.5	3.5	0.4
Zn/mg·kg^−1^	49.27	176.79	96.56	96.56	ND
Cd/mg·kg^−1^	1.21	1.19	2.26	2.26	ND
As/mg·kg^−1^	55.89	51.99	1.43	1.43	ND
Cu/mg·kg^−1^	365.10	1067.82	18.37	18.37	ND

Note. ND means not found.

**Table 3:** General features of strains *M. rubeus* P1, P2, and P3 and *M. kameinonensis* P4.

Property	*M. kameinonensis* P4	*M. rubeus* P3	*M. rubeus* P2	*M. rubeus* P1	*M. pedocola* sp. TBZ30^T^
Gram strain	Negative	Negative	Negative	Negative	Negative
Cell shape	Rod-shaped	Rod-shaped	Rod-shaped	Rod-shaped	Rod-shaped
Colony colour	Light-yellow	Pink	Pink	Pink	Pink
pH	5.0–7.0 (5.0)	5.0–9.0 (5.0)	5.0–8.0 (5.0)	5.0–8.0 (6.0)	5.0–8.5 (7.0)
Temperature range (°C)	4–37 (28)	4–37 (28)	4–37 (28)	4–37 (28)	4–28 (25)
Oxygen requirement	Aerobic	Aerobic	Aerobic	Aerobic	Aerobic
Salinity (%)	0–1.5 (0)	0–1.0 (0)	0–1.5 (0)	0–1.0 (0)	0–1.0 (0)

**Table 4:** Heavy metal related genes in the analyzed *Mucilaginibacter* strains in comparison to *Cupriavidus metallidurans* CH34.

Genes Encoding Heavy Metal Resistance Determinants		*M. kameinonensis* P4	*M. rubeus* P3	*M. rubeus* P2	*M. rubeus* P1	*C. metallidurans* CH34
P_1B_-type-ATPase	P_IB-1_ type-ATPase	3	6	6	6	7
	P_IB-3_ type-ATPase	1	4	5	4	0
	P_IB-4_ type-ATPase	2	2	4	4	1
	Mg^(II)^	0	1	1	1	0
RND type metal transport systems	CzcCBAD	8	10	10	11	9
	CusCBA	0	2	4	3	2
	NccCBA	0	0	2	2	1
*ars* operons		2	3	4	4	1
Multicopper oxidases		2	6	5	6	2

RND: Resistance-nodulation-division.
